# Natural compounds: new therapeutic approach for inhibition of *Streptococcus mutans* and dental caries

**DOI:** 10.3389/fphar.2025.1548117

**Published:** 2025-04-01

**Authors:** Milad Kashi, Mahdieh Varseh, Yasaman Hariri, Zahra Chegini, Aref Shariati

**Affiliations:** ^1^ Student research Committee, Arak University of Medical Sciences, Arak, Iran; ^2^ Student Research Committee, Khomein University of Medical Sciences, Khomein, Iran; ^3^ Department of Microbiology, School of Medicine, Hamadan University of Medical Sciences, Hamadan, Iran; ^4^ Infectious Diseases Research Center (IDRC), Arak University of Medical Sciences, Arak, Iran

**Keywords:** natural compounds, *Streptococcus mutans*, biofilm, dental caries, new treatment

## Abstract

*Streptococcus mutans* is recognized as one of the leading causes of dental caries, and biofilm formation by this bacterium plays a key role in dental plaque development and caries progression. Given the increasing resistance of bacteria to antibiotics and the adverse effects of some synthetic antimicrobials, the search for natural alternatives has received increasing attention. The recently published studies have demonstrated that natural compounds (NCs) such as curcumin, cinnamaldehyde, eugenol, thymol, carvacrol, epigallocatechin gallate, farnesol, catechin, inulin, menthol, apigenin, myricetin, oleanolic acid, and resveratrol, have notable antimicrobial properties and can effectively inhibit the growth of *Streptococcus mutans*. NCs can disrupt bacterial membrane integrity, leading to cell death, and possess the capability to inhibit acid production, which is a key factor in caries development. NCs can also interfere with bacterial adhesion to surfaces, including teeth. The attachment inhibition is achieved by decreasing the expression of adhesion factors such as *gtf*s, *ftf*, *fruA*, and *gbpB*. NCs can disrupt bacterial metabolism, inhibit biofilm formation, disperse existing biofilm, and interfere with quorum sensing and two-component signal transduction systems. Moreover, novel drug delivery platforms were used to enhance the bioavailability and stability of NCs. Studies have also indicated that NCs exhibit significant efficacy in combination therapies. Notably, curcumin has shown promising results in photodynamic therapy against *S. mutans*. The current review article analyzes the mechanisms of action of various NCs against *S. mutans* and investigates their potential as alternative or complementary therapeutic options for managing this bacterium and dental caries.

## Introduction

Dental caries is a prevalent chronic infectious disease affecting the teeth’ hard tissues. Dental caries and its sequelae can aggravate or trigger systemic disorders, significantly diminishing the quality of human life. Dental caries has a complicated etiology; two primary causative variables are the regular intake of free sugars and the metabolic activity of certain commensal, tooth-adherent bacteria. Numerous oral microbes generate organic acids through the metabolism of fermentable carbohydrates. These acids lower salivary pH, leading to the demineralization of tooth tissue. A notable diversity of microbe species linked to dental caries has been identified, including *Streptococcus mutans*, the most prevalent bacterium observed in individuals with dental caries (Bhaumik et al., 2021).

This bacterium is a Gram-positive, facultative anaerobic, and catalase-negative bacterium that generates lactic acid and can lower the environmental pH from seven to 4.2 within approximately 24 h. Furthermore, it can ferment and create acids from carbohydrates, including glucose, lactose, and raffinose, which is why it is identified as the primary pathogen in the onset of dental caries. This bacterium can degrade carbohydrates and synthesize glucan, which is crucial for interacting with dental structures (Lemos et al., 2019). Carbohydrates like sucrose are metabolized to create intracellular and extracellular polysaccharides. The synthesis of extracellular polysaccharides (EPS) facilitates bacterial adherence and accumulation on the tooth surface, resulting in structural alterations such as enhanced porosity in dental biofilms (Liu et al., 2018; Lemos et al., 2019).

To endure the challenging conditions of the human oral cavity, *S. mutans* and other oral microorganisms establish a highly structured microbial consortium known as a biofilm (Marsh and Zaura, 2017). Biofilm is a complex structure composed of aggregated microbial cells and microbially produced extracellular polymeric substances (Marsh and Zaura, 2017).

Following initial adhesion, bacteria such as *S. mutans* begin to grow and synthesize extracellular polymeric substances, forming a stable three-dimensional community that incorporates pathways to distribute nutrients, oxygen, and signalling chemicals efficiently. This bacterium substantially enhances biofilm formation via both sucrose-dependent and sucrose-independent mechanisms. The sucrose-dependent mechanism mostly depends on the extracellular glucose transferase. *Streptococcus mutans* secretes three extracellular glucose transferases: GtfB, GtfC, and GtfD. GtfB primarily generates viscous, water-insoluble polysaccharides from sucrose, whereas GtfC produces a combination of insoluble and soluble polysaccharides from sucrose. GtfD mainly catalyzes the conversion of sucrose into soluble polysaccharides. The sucrose-independent mechanism involves contact between the sticky particles of *S. mutans* and the acquired enamel pellicle. Agglutinins present in saliva facilitate the adherence and aggregation of *S. mutans* by their interaction with the I/II antigen, a multifunctional PI adhesin that is anchored in the bacterial cell wall and encoded by the *spaP* gene (Bowen and Koo, 2011; Krzyściak et al., 2014).

Numerous techniques exist for addressing oral and dental ailments, including non-surgical and surgical interventions and adjuvant therapies such as antibiotics. Although antibiotics are helpful, excessive dependence on them might result in the emergence of resistant bacterial strains, diminishing their potency over time. Furthermore, broad-spectrum antibiotics can disturb the normal equilibrium of the oral microbiota, potentially resulting in opportunistic infections or other oral health complications (Serino et al., 2001; Ryan, 2005). To this end, certain plants without undesirable side effects have demonstrated more efficacy than manufactured medications in preventing dental caries (Malvania et al., 2019). The natural compounds derived from various plant components, including roots, leaves, and fruits, have distinct medical effects when subjected to alterations (Rangel et al., 2018). Most antibacterial compounds in plants often encompass flavonoids, phenols, alkaloids, and organic acids (Shad et al., 2014). Multiple studies have indicated that managing dental biofilm is essential for preventing tooth decay. Quorum sensing (QS) is a crucial virulence regulator in cariogenic biofilms. Biofilm generation relies on the signal-mediated QS system. Plant extracts can block QS genes, disrupting biofilm formation (Choi et al., 2017; Balhaddad et al., 2019). In addition, these compounds also inhibit glucosyltransferase, which is crucial in creating water-insoluble glucan, preventing the development of cariogenic biofilms (Yabuta et al., 2018; Farkash et al., 2020).

Because of its consistently high frequency and exorbitant treatment costs, dental caries continues to be a significant problem with significant long-term health, economic, and societal effects (Hescot, 2017; Kassebaum et al., 2017). To this end, finding new therapeutic approaches for managing *S. mutans*-associated dental caries is necessary. In the present review article, we will discuss the interactions of natural compounds with *S. mutans* to improve the scientists’ knowledge of using these compounds for managing dental caries.

## Curcumin

Curcumin is an orange-yellow pigment in the rhizome of *Curcuma longa* and demonstrates a broad spectrum of medicinal actions, including antibacterial and antiseptic properties (Supplementary Table S1) (Kashi et al., 2024). Studies have investigated and confirmed the antibacterial properties of curcumin against *S. mutans* (Song et al., 2012; Hu P. et al., 2013). For instance, one study reported that curcumin’s minimum inhibitory concentration (MIC) against *S. mutans* was 64 μM (Ke et al., 2023). This bacterium metabolizes carbohydrates, resulting in medium acidity. *Streptococcus mutans* markedly alters the fatty acid composition of its membrane in response to ambient acidity, implicating fatty acid metabolism (Baker et al., 2017). Curcumin can affect fatty acid, carbon and pyruvate metabolism in *S. mutans*. These metabolic pathways are crucial for the bacteria’s survival as they are its principal energy sources (Guzmán-Flores et al., 2024). Curcumin also influences DNA replication and the metabolism of purines and pyrimidines in *S. mutans*. Modifying these metabolic pathways will likely lead to bacterial mortality (Guzmán-Flores et al., 2024). Notably, a primary virulence component of *S. mutans* is the unique lipid composition of its membrane, which varies with pH; thus, curcumin modulates the molecules implicated in the lipid response (Guzmán-Flores et al., 2024).

As mentioned, *S. mutans* metabolises carbohydrates, generating an acidic microenvironment in tooth plaque. Therefore, this bacterium must have a mechanism for enduring acidic environments. The enzyme F-ATPase regulates cytoplasmic pH by extruding protons from the cytoplasm in acidic environments (Sekiya et al., 2019). A study reported that the expression of the F-ATPase gene in *S. mutans* was increased in acidic environments (Kuhnert et al., 2004). However, researchers proposed that this enzyme is non-essential for bacterial proliferation under neutral circumstances. Curcumin significantly diminished the ATPase activity of *S. mutans* F-ATPase and suppressed the proliferation of this bacterium at pH 5.3. The data demonstrated that *S. mutans* exhibited significant sensitivity to F-ATPase inhibitors in acidic environments, highlighting the critical function of F-ATPase in the acid tolerance of this bacterium (Sekiya et al., 2019). Moreover, a study revealed that the *atpH* gene, which encodes subunit C of the F-ATPase enzyme, was downregulated in *S. mutans* following curcumin treatment (Li et al., 2018). Thus, curcumin can impede the acid tolerance capacity of S*. mutans* and likely diminish its cariogenic characteristics (Galvão et al., 2015). Alongside acid stress, oxidative stress constitutes a primary environmental obstacle encountered by *S. mutans* in the oral environment (Galvão et al., 2015). This bacterium exhibited susceptibility to hydrogen peroxide (H_2_O_2_), which is generated through the metabolic processes of other species in dental plaque (Higuchi et al., 1999). Exposure to elevated concentrations of H_2_O_2_ and its deleterious byproducts, hydroxyl and superoxide anions, can induce irreversible cellular damage. Spx, comprising two homologues, SpxA1 and SpxA2, regulates the transcription of nearly all principal activated oxidative stress response genes in *S. mutans* (Baker et al., 2014; Kajfasz et al., 2017; Ganguly et al., 2020). *Ke* et al. found that curcumin treatment increased SpxA1 and SpxA2 levels, perhaps leading to increased H_2_O_2_ generation through the activation of oxidative stress response genes (Ke et al., 2023). All these effects that occur in the bacteria when exposed to curcumin can lead to growth inhibition and cell death.

In addition, curcumin can impede biofilm formation and markedly affect the biofilm development of *S. mutans* (Ke et al., 2023). This compound diminished the quantity of viable and total bacteria within the biofilm and decreased the biofilm’s thickness. The diminution in biofilm thickness would result in the alteration of the three-dimensional structures of the biofilm, potentially impacting the cariogenicity of *S. mutans* (Li B. et al., 2020). Furthermore, curcumin prevented the biofilm development of *S. mutans* by disrupting EPS (Bottner et al., 2020). This compound can significantly reduce the amounts of EPS in biofilm and destroy the structure of EPS in the short term, decreasing the EPS biomass (Li et al., 2018; Li et al., 2019; Li B. et al., 2020). Furthermore, the expression of *gbpB* was downregulated after curcumin treatment (Li et al., 2018; Li B. et al., 2020). Bacterial aggregation is a crucial factor in the production of EPS, facilitated by interactions among surface-associated glucan-binding proteins (GbpBs) that stick to glucan, thus enhancing plaque formation (He et al., 2012). The absence or mutation of the gene encoding GbpB leads to alterations in cell morphology and a deceleration of growth. The mentioned change inhibits the proper formation of biofilm, which results from irregular cell clusters encased in a matrix of atypical structure (Duque et al., 2011). The inhibitory effect of curcumin on EPS and GbpB can significantly decrease biofilm formation.

Curcumin can downregulate the expression of *rgpG*, *scrAB*, *gtfB*, *gtfC*, *gtfD*, *ftf* and *fruA* (Li et al., 2018; Li et al., 2019; Li B. et al., 2020; Ke et al., 2023). As previously mentioned, the Gtfs are essential enzymes for bacteria to utilize sucrose and form glucan. Reduced *gtfB*, *gtfC*, and *gtfD* expression disrupts GbpB-mediated bacterial aggregation (Li B. et al., 2020). RgpG is a protein associated with the cell envelope that is implicated in the biofilm development of *S. mutans* (De et al., 2017). A loss in the *rgpG* gene leads to a significant decrease in cell surface antigens and substantial abnormalities in cell shape and division without affecting growth (De et al., 2017; Bischer Andrew et al., 2020). RgpG-deficient mutants showed elongated chains of inflated “Square” developing cells and produced less biofilms irrespective of the carbohydrate source. Curcumin downregulated *rgpG*, indicating that it influences cell division and diminishes biofilm formation via targeting *rgpG* (De et al., 2017). Furthermore, both *ftf* and *fruA* expressions were dysregulated following short-term exposure to curcumin. Fucosyltransferase (FTF) is an enzyme that catalyzes the conversion of sucrose into extracellular homopolymers of fructose, known as fructans. FTF, derived from the *fruA* gene, is an exo-β-d-fructosidase that liberates fructose from β(2.6)- and β(2.1)-linked fructans, as well as cleaving fructose from sucrose and raffinose (Li et al., 2018). Consequently, the quantity of fructans synthesized by the bacterium is markedly diminished after treatment with curcumin. This reduction diminished the bacterium’s capacity to adhere, hindering its effective utilization of sucrose as an energy source (Li et al., 2018). There is a decreasing tendency in the VicR expression of *S. mutans* after curcumin treatment (Li et al., 2018). VicR functions as a response regulator by binding to the promoter regions of the *gtfB*, *gtfC*, and *ftf* genes, thereby activating these genes and promoting biofilm formation (Senadheera et al., 2005). Therefore, the reduction of VicR negatively impacts the biofilm formation process. In the end, the *scrA* gene in the phosphotransferase system (PTS) of *S. mutans* encodes a high-affinity permease that facilitates the internalization of sucrose. Subsequently, intracellular sucrose-6-phosphate is initially hydrolyzed by ScrB, a sucrose-6-phosphate hydrolase, yielding fructose and glucose-6-phosphate (Li et al., 2018). The *scrAB* genes were downregulated in the presence of curcumin, diminishing the availability of sucrose and its derivatives within the cell. This alteration impacts biofilm formation, as sucrose is a primary energy source and nutrient for establishing *S. mutans* biofilms.

QS systems play a crucial role in regulating biofilm development and activating virulence factors in various bacteria, making them an attractive target for combating biofilm infections. Two extensively researched QS systems exist in *S. mutans*: the competence-stimulating peptide-QS (CSP-QS) system, which comprises a competence-stimulating peptide (encoded by *comC*), a histidine kinase sensor protein (encoded by *comD*), and a cognate response regulator (encoded by *comE*). This system facilitates intraspecies cell-cell communication and positively regulates the expression of biofilm-related genes such as *gtfB*, *gtfC*, and *gbpB* in *S. mutans*. The second system is the LuxS system, which catalyzes the synthesis of the signaling peptide Autoinducer 2 (AI-2) to mediate interspecies and intraspecies interactions within the multispecies plaque community (Li et al., 2018; Li et al., 2019). To this end, recently published studies reported that curcumin may reduce the number of viable bacteria in the biofilm and diminish the biomass of EPS in *S. mutans* biofilm by suppressing the expression of the LuxS system and ComCDE system (Li et al., 2018; Li et al., 2019).

Consequently, as previously stated, curcumin has demonstrated encouraging efficacy against *S. mutans*. Nonetheless, the low water solubility, inadequate stability, rapid clearance rate, and restricted bioavailability of curcumin have constrained its clinical utilization (Hu et al., 2023). In recent years, nanodrugs delivery technologies have been extensively employed across diverse domains. Nanoparticles can be engineered to induce drug release under various conditions, ensuring that drugs remain unaffected by pH, enzymes, and other variables. Nanoparticles effectively enhance the accessibility of water-insoluble or sparingly soluble bioactive compounds. *Hazzah* et al. synthesized and analysed curcumin solid lipid nanoparticles (CurSLN) to manage oral mucosal infections. CurSLN had superior antibacterial efficacy to raw curcumin and chemically stabilized curcumin, demonstrating a MIC of 0.09375 mg/mL against *S. mutans* (Hazzah et al., 2015). In another study, a bio-nanocomposite comprising carboxymethyl starch (CMS), chitosan (CS), and montmorillonite (MMT) was formulated for the delivery of curcumin. The curcumin-loaded bionanocomposite significantly inhibited biofilm growth in dental models (Jahanizadeh et al., 2017). Moreover, researchers assessed the mechanical characteristics and antibacterial efficacy of varying doses of Curcumin-Nisin-poly (L-lactic acid) nanoparticles (CurNisNps) incorporated into orthodontic acrylic resin against *S. mutans* and *Candida albicans*. The study results demonstrated significant anti-biofilm efficacy of the synthesized platform against microorganisms throughout a 60-day follow-up period. Furthermore, CurNisNps substantially diminished the expression levels of *gtfB* (Pourhajibagher et al., 2022). Hence, in recent years, nanotechnology has revolutionized the properties of materials. Researchers have increased its pharmacokinetics properties and antimicrobial activity by incorporating curcumin into nanocarriers, leading to more effective infection treatments.

**FIGURE 1:**
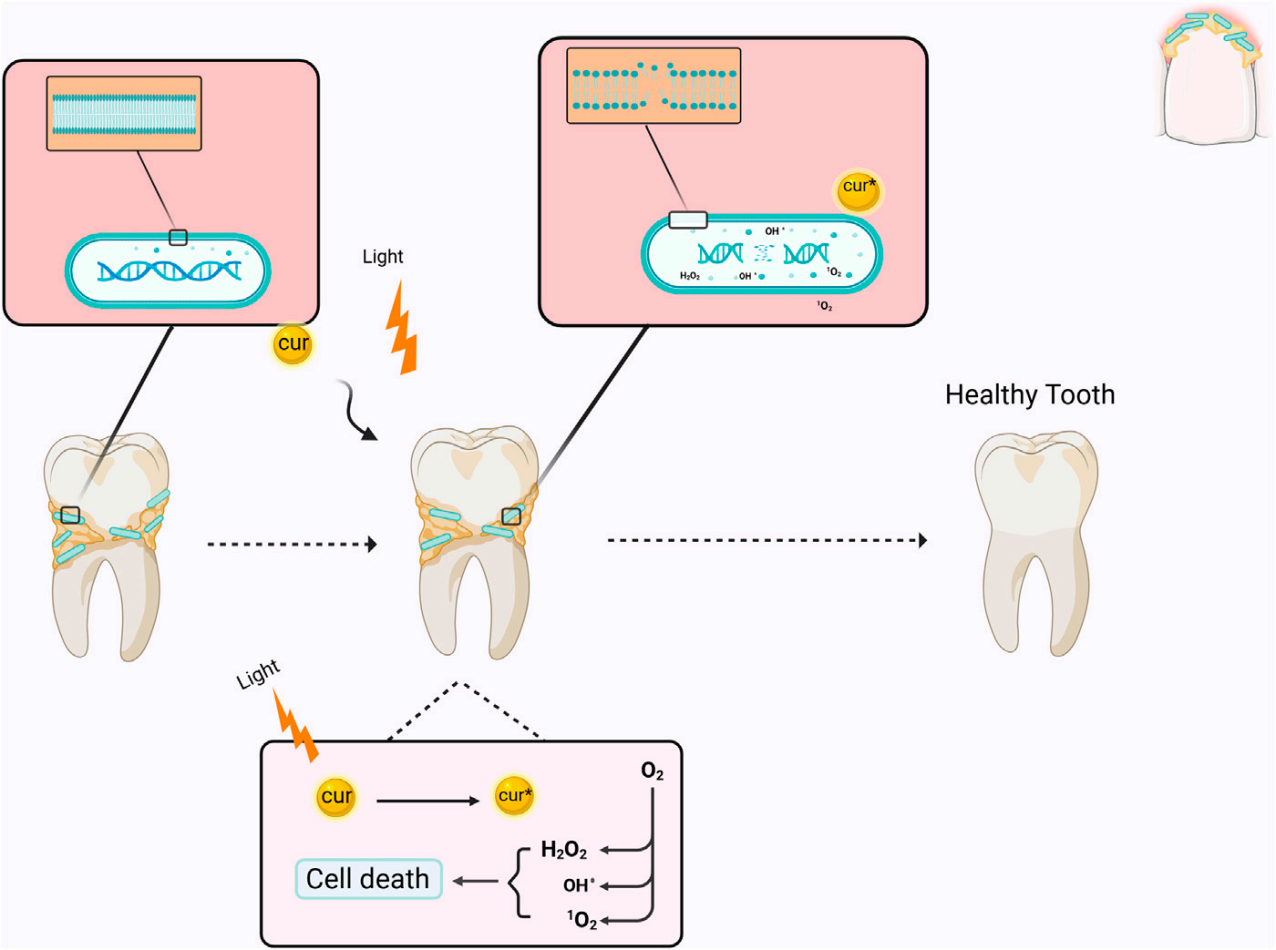
Schematic illustration of the mechanism of curcumin as a photosensitizer in PDT. When curcumin is excited by light irradiation, it converts O_2_ into reactive oxygen species, which causes bacterial death by affecting the membrane and DNA. Cur: curcumin.

Photodynamic therapy (PDT) is a therapeutic approach that utilizes visible light with photosensitizers (PS) or dyes. The PS attaches to the target cell membrane and, when exposed to visible light at a specific wavelength (unique to each PS), induces the generation of various reactive oxygen species (ROS), including singlet oxygen, which triggers a cascade of biological events resulting in apoptosis of cells or the death of microorganisms (Paulino et al., 2005; Paschoal et al., 2015). Curcumin predominantly absorbs light in the wavelength range of 400–500 nm; therefore, a suitable light source must be utilized when employing curcumin as a PS (Figure 1) (Table 1) (Paschoal et al., 2013). The application of curcumin in an *in vivo* study revealed no burning sensation, mouth discomfort, or ulcers (Araújo et al., 2012b). In this regard, a study examined the differential impacts of nanomicelle curcumin-based PDT (NMCur-aPDT) on the microbial population and pathogenicity of *S. mutans*. The results indicated that the antibacterial and anti-virulence efficacy of NMCur-aPDT against *S. mutans* surpassed that of the other treatment groups, and the gene expression level of *gtfB* diminished (Hosseinpour-Nader et al., 2022). Moreover, *Hu* et al. engineered a liposome with adhesive characteristics to transport curcumin into the biofilm. This research demonstrated that curcumin can be released from the liposome near the biofilm, exerting an antibacterial impact by dispersing the biofilm under blue light irradiation (Hu et al., 2023). Therefore, PDT, curcumin, and novel drug delivery systems provide an innovative approach to treating bacterial infections, especially *S. mutans*.

**TABLE 1:** Curcumin-based photodynamic therapy for inhibition of *Streptococcus mutans*.

Study model	Bacteria	Light source	Outcome	References
PDT using curcumin	*S. mutans* and *Lactobacillus acidophilus*	Blue light (450 nm)	PDT was effective in reducing bacteria in planktonic cultures	Araújo et al. (2012a)
Photodynamic antimicrobial effect of curcumin	*S. mutans* ATCC 700610	White light (42 J/cm^2^)	The PACT groups demonstrated a bacterial decrease exceeding 5 log10 relative to the control	Paschoal et al. (2014)
PDT using curcumin	*S. mutans* and *L. acidophilus*	LED (5.7 J/cm^2^)	A remarkable decrease in cell viability was detected in the biofilm community	Araújo et al. (2014)
PDT using curcumin	*S. mutans* ATCC 2517 and clinical isolate	Blue LED (450 nm)	PDT showed a significant antibacterial effect on *S. mutans* standard strain and a less pronounced impact on its clinical isolate	Tonon et al. (2015)
Photoinactivation effect of curcumin	*S. mutans*, *Streptococcus sanguinis* and *Candida albicans*	White light (90 mW/cm^2^)	It was effective in killing *S. mutans* and *S. sanguis* strains but ineffective against *C. albicans*	Soria-Lozano et al. (2015)
Curcumin-mediated API	*S. mutans*, *C. albicans*, and *Candida glabrata*	LED (37.5 J/cm^2^)	Both 24h and 48 h biofilms were susceptible to API.	Quishida et al. (2016)
aPDT with curcumin	*S. mutans* ATCC 25175	LED (25.3 J/cm^2^)	The viability of *S. mutans* in the presence of curcumin was substantially reduced during irradiation	Lee et al. (2017)
Curcumin as a photosensitizing dye	*S. mutans*	Blue-violet diode (405 nm)	The treatment inhibited the growth of bacteria up to 99.26%	Merigo et al. (2019)
PDT using curcumin	*S. mutans* ATCC str.m 1,683	Laser (460 and 660 nm)	460 nm laser + curcumin had the most significant effect on inhibiting the growth of *S. mutans* bacterial colonies	Azizi et al. (2019)
Curcumin as a PS agent in aPDT.	*S. mutans* ATCC 700610	LED (1.2 J/cm^2^)	The treated group showed a reduction in viability compared to the control	Sanches et al. (2019)
Curcumin-mediated PDI with EDTA	*S. mutans* UA159	Blue LED (33.5 J/cm^2^)	PDI showed a strong inhibitory effect against *S. mutans* in planktonic culture	Nima et al. (2021)
aPDT with curcumin-loaded dental resin	*S. mutans* ATCC 700610	Blue light (14.6 J/cm^2^)	A 2 log_10_ (CFU/mL) reduction in *S. mutans* was observed after light application on the biofilms	Comeau et al. (2022)
aPDT using curcumin as a photosensitizer	*S. mutans*	Blue diode laser (445 nm)	It can effectively reduce colonies of *S. mutans* around stainless steel brackets	Pordel et al. (2023)
aPDT with using curcumin	*S. mutans* ATCC 35668	Blue LED (450 nm)	aPDT caused a significant reduction in the viability of *S. mutans* in both planktonic and biofilm forms	Ahrari et al. (2024)

PDT: photodynamic therapy. aPDT: antibacterial photodynamic therapy. PDI: photodynamic inactivation. PS: photosensitizer. API: antimicrobial photodynamic inactivation. PACT: photodynamic antimicrobial chemotherapy.

Curcumin has shown significant antibacterial activity against *S. mutans*, influencing bacterial metabolic pathways and reducing its acid tolerance capacity. This compound also possesses anti-biofilm properties that can affect all stages of biofilm formation. As a photosensitizer, curcumin exerts its antibacterial effects by generating ROS. However, considering its limited pharmacokinetic properties, innovative drug delivery platforms can be employed to enhance its effectiveness.

## Cinnamaldehyde

**FIGURE 2:**
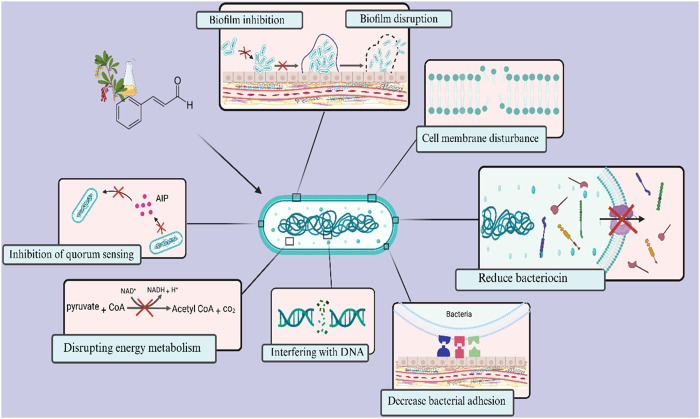
Overview of antimicrobial targets of cinnamaldehyde against *Streptococcus mutans*. Cinnamaldehyde primarily possesses the ability to penetrate cellular membranes and alter their physicochemical properties. Additionally, it can disrupt the energy metabolism and ATP production in bacteria. Furthermore, it diminishes bacterial survival by influencing the expression of genes associated with bacteriocin production and DNA replication and maintenance. This compound also impacts the formation and maturation of biofilms, affecting critical stages such as surface adhesion and intercellular communication through the quorum sensing system.

Cinnamaldehyde is a bioactive chemical extracted from cinnamon bark, recognized for its varied effects, including antifungal and antibacterial characteristics (Kashi et al., 2024). Researchers have lately shown interest in utilizing the antibacterial properties of cinnamaldehyde to tackle *S. mutans* (Sharma et al., 2016; de Almeida et al., 2020; Ngokwe et al., 2024). In one study, the minimum bactericidal concentration (MBC) values of trans-cinnamaldehyde against planktonic *S. mutans* were reported to be 1728 μg/mL. At this concentration, trans-cinnamaldehyde also caused a 50% reduction in biofilm metabolic activity (Ribeiro et al., 2018). The hydrophobic properties of cinnamaldehyde facilitate its contact with the cell membrane of *S. mutans* (Ribeiro et al., 2018). Furthermore, the topological polar surface area (TPSA) < 40 Å^2^ reported for cinnamaldehyde suggests it may possess a favourable capacity for permeating cell membranes, as only compounds with TPSA >140 Å^2^ often exhibit poor permeability (Veber et al., 2002). Highly hydrophobic chemicals are typically associated with increased toxicity, and the cytoplasmic membrane frequently serves as the principal target for antimicrobial activity. Lipophilic substances exhibit a strong affinity for cell membranes *via* altering the physicochemical properties of the membrane (Figure 2) (Ribeiro et al., 2018).

Cinnamaldehyde can interfere with carbohydrate metabolism, glycolysis, pyruvate metabolism, and the tricarboxylic acid (TCA) cycle, as well as arginine, tryptophan, and proline metabolism of *S. mutans*. Researchers speculate that the primary mechanism of action of cinnamaldehyde against *S. mutans* is targeting pyruvate dehydrogenase (PDH), which affects its downstream TCA cycle pathway, thereby inhibiting energy production. This energy level reduction impacts the bacteria’s upstream carbohydrate metabolism and the glycolytic pathway. Additionally, amino acid metabolism is affected (Zhang et al., 2024). Noteworthy, the TCA cycle is critical in energy metabolism, and PDH is one of the key enzymes entering the TCA cycle. In *S. mutans*, the TCA cycle has been proven incomplete, and its principal significance is in producing intermediates for other metabolisms (Zhang et al., 2024). By acting on PDH, cinnamaldehyde interferes with the downstream TCA cycle, which could not further generate other metabolic intermediates. The pyruvate-related genes (*pdhA*, *pdhB*, *pdhC*, and *pdhD*), which encode different subunits of the PDH enzyme complex, were downregulated after the treatment of cinnamaldehyde (Zhang et al., 2024). Additionally, expression of the *atpD* gene was downregulated in the presence of cinnamaldehyde (Balasubramanian et al., 2021). The *atpD* gene in *S. mutans* encodes the β-subunit of the ATP synthase enzyme, which is essential for cellular energy production (Gabe et al., 2019). Therefore, cinnamaldehyde can disturb cell membranes and inhibit energy metabolism and ATP production of *S. mutans* (Zhang et al., 2024).

*Streptococcus mutans* has a strong ability to acid production (acidogenicity) and acid resistance (acidurance), and it can convert glucose into lactic acid and other acidic substances by glycolysis (He et al., 2019; Zhang et al., 2024). The findings of recently published studies showed that cinnamaldehyde suppressed glucose and sucrose consumption in *S. mutans* and downregulated the expression levels of glycolysis-related genes, such as *eno*, *ldh*, and *pykf* (He et al., 2019; Zhang et al., 2024). Under excess carbohydrate and oxygen deficiency conditions, *S. mutans* tend to convert pyruvate to lactic acid by lactate dehydrogenase (LDH) and convert NADH to NAD again. The accumulation of lactic acid decreases the pH (Abranches et al., 2018). With the cinnamaldehyde interfering with the glycolysis pathway and reducing the LDH activities, the acid production is inhibited, and then the terminal pH increases (He et al., 2019; Zhang et al., 2024). DNA repair processes are enhanced in response to acidogenicity to sustain homeostasis (Senadheera et al., 2009). The genes *atpD*, *dnaK* (associated with stress tolerance), and the DNA repair mechanisms *idh* and *recA* were consistently downregulated, inhibiting stress tolerance in the presence of cinnamaldehyde (Balasubramanian et al., 2021). In addition, *relA* gene expressions were downregulated by cinnamaldehyde (He et al., 2019). Guanosine tetra (penta)-phosphate synthetize/hydrolase is encoded by the *relA* gene, and it is implicated in various processes, including acid and oxidative stress tolerance mechanisms, as well as biofilm production (Lemos José et al., 2004). Furthermore, cinnamaldehyde can decrease the expression of the *brpA* gene. It has a role in cell wall integrity, which is important to deal with physical and chemical stresses in the environment. The *brpA* also is known to control *recA*, *dnaK*, and *atpD* (Balasubramanian et al., 2021). As a result, the survival rate of bacteria in an acidic environment and acid resistance of *S. mutans* gradually decreased after treatment with cinnamaldehyde (He et al., 2019; Zhang et al., 2024).

Bacteriocin immunity proteins are crucial for conferring immunity to bacterial cells. Bacteriocin immunity proteins-related genes, such as *immA* and *immB*, nisin-like mutacin C (*nlmC*), and *bsmI*, were downregulated by cinnamaldehyde (Balasubramanian et al., 2021). The *atlA* gene also was downregulated by cinnamaldehyde (Balasubramanian et al., 2021). This gene encodes an autolysin enzyme essential for cell wall biogenesis, facilitating cell growth and division in *S. mutans* (Ahn and Burne, 2006). In the end, a significant downregulation of *ftsZ* and *gyrA* was detected after cinnamaldehyde treatment, suggesting that this compound affects the development and metabolism of *S. mutans*. The ***gyrA*** gene plays a critical role in DNA replication and maintenance of genomic stability, and ***ftsZ*** is essential in cell division (Marchese and Debbia, 2016; Barrows and Goley, 2021). Thus, cinnamaldehyde can compromise the viability of *S. mutans*.

The interactions between the cinnamaldehyde and *S. mutans* biofilm community are also interesting. The results of the studies showed that *S. mutans* biofilms were highly dispersed and visibly loose, and there was an irregular distribution of biofilm with reduced biomass following cinnamaldehyde treatment, which can be readily eradicated by host clearance mechanisms (He et al., 2019; Balasubramanian et al., 2021). *He* et al. reported that cinnamaldehyde might attenuate biofilm development during the initial adhesion and maturation stages. Moreover, this compound enhanced the hydrophobicity and reduced the aggregation of *S. mutans* (He et al., 2019). A decrease in the attachment and aggregation of bacteria can be related to the downregulation of *gtfB*, *gtfC*, *gtfD*, *gbpB*, *spaP* and *ftf* genes (He et al., 2019; Balasubramanian et al., 2021; Zhang et al., 2024). Also, the *ciaH*, *covR* and *vicR* genes were downregulated by cinnamaldehyde (He et al., 2019). CiaRH is a significant two-component signal transduction systems (TCSTS) associated with biofilm development, acid tolerance, and genetic competence. The removal of the *ciaH* gene impacts bacterial adhesion, decreases sucrose-independent biofilm formation, eliminates mutacin synthesis, and reduces competence development (Wu et al., 2010). Moreover, the VicRK system and the orphan regulator CovR of *S. mutans* collaboratively regulate virulence genes. VicR and CovR directly regulate a set of genes, including *gtfB*/C/D and *gbpC,* which are involved in the synthesis and interaction with extracellular polysaccharides; alterations in their expression can affect attachment and biofilm formation (Stipp et al., 2013). Finally, cinnamaldehyde also affects the QS system. It can downregulate the expression level of QS-related genes, including *comA*, *comE*, *comS*, *comR*, *comDE*, *comB*, *comEC*, and *luxS* (Balasubramanian et al., 2021; Zhang et al., 2024). The downregulation of *luxS* may lead to the downregulation of *brpA*, *comDE, vicR*, *recA*, and *spaP* (Wen et al., 2011). Therefore, cinnamaldehyde can interfere with biofilm formation by *S. mutans* in various ways.

Researchers have devised many techniques to administer cinnamaldehyde to enhance its bioavailability *in vivo* (Zaltsman et al., 2017; Jailani et al., 2022; Mu et al., 2023). A study investigated the efficacy of trans-cinnamaldehyde (TC) encapsulated in porous silicon (pSi) particles in inhibiting biofilm formation. The results indicated that pSi-TC at concentrations of 0.5 mg/mL and 1.15 mg/mL reduced the development of *S. mutans* biofilm by 78% and 85%, respectively. The synthesized particles decreased lactic acid production by 25.11% in biofilms and significantly downregulated the *S. mutans* genes responsible for inter-species communication and biofilm development (*luxS*), genes that regulate heat and acid-induced stress (*dnaK* and *atpD*), and oxidative stress tolerance (*nox1*). Moreover, pSi-TC markedly downregulated the genes encoding glucosyl transferases (*gtfB* and *gtfC*) (Jailani et al., 2022). In another study, a nanosystem designed to combat caries was developed by encapsulating cinnamaldehyde within chitosan-based nanocapsules (CA@CS NC). The authors proposed that CA@CS NC can adsorb the bacterial membrane through electronic interactions and release cinnamaldehyde over an extended period. Simultaneously, the nanoparticles demonstrated consistent antibacterial efficacy against *S. mutans* and reduced the expression levels of QS, virulence, biofilm, and adhesion genes, including *gtfB*, *gtfC*, and *gtfD* (Mu et al., 2023). Consequently, using nanoparticles to transport cinnamaldehyde presents novel opportunities to enhance its application in creating antibacterial and antibiofilm agents.

## Eugenol

Eugenol, or 4-allyl-2-methoxyphenol, is a fragrant oily liquid derived from certain essential oils, notably clove and cinnamon. It has been a flavoring ingredient in culinary and cosmetic preparations (Didehdar et al., 2022). Eugenol has shown good antibacterial activity against *S. mutans*. In one study, its MBC value against this bacterium was 1,642 μg/mL (Ribeiro et al., 2018). This compound, like cinnamaldehyde, has a hydrophobic state that allows it to interact with the cell membrane of *S. mutans* (Ribeiro et al., 2018). In addition, a TPSA of <40 Å^2^ has been reported for this compound, suggesting it can penetrate the cell membrane (Veber et al., 2002). Eugenol impeded the decrease in pH caused by *S. mutans*. The findings indicated that eugenol can diminish acid generation by *S. mutans* (Xu et al., 2013). As previously mentioned, acidogenicity is one of the important factors associated with dental caries, and its disruption by eugenol reduces its activity for demineralization.

The antibiofilm efficacy of eugenol is evidenced by the decrease in the proportion of biofilm formation in its presence. Biofilms treated with eugenol exhibited deformed cellular structures, indicating the significant effect of eugenol on cell architecture and cellular damage, perhaps leading to the release of intracellular material. Significantly, biofilm developed in the presence of eugenol exhibited reduced cell aggregation and biofilm disorganization, with a notable reduction in colony-forming units (CFU) and matrix production (Jafri et al., 2019; Jafri et al., 2020). A further consideration is the synthesis of water-insoluble glucans by *S. mutans*, which was inhibited in the presence of eugenol (Xu et al., 2013). Water-insoluble glucans promote the adhesive interactions of bacteria with the tooth surface and contribute to the formation of dental biofilms (Xu et al., 2013). Additionally, the genes that affect bacterial adhesion and biofilm formation include *gtfB*, *gtfC*, *gbpB*, *ftf*, *vicR*, *brpA*, *smu630*, *relA, comDE*, and *spaP* were downregulated by eugenol (Adil et al., 2014). With all these interpretations, it can be concluded that eugenol can affect the *S. mutans* biofilm, making it a promising natural agent for combating dental biofilms.

Eugenol can be utilized alongside antibacterial agents to achieve synergistic or additive effects. The synergistic effects of eugenol and carnosic acid on microbial biofilms, specifically *S. mutans*, were noted. The interactions of eugenol and carnosic acid with the cytoplasmic membrane may elucidate this phenomenon. The combination of eugenol and macrocarpals exhibited synergistic benefits against bacterial biofilms. The rupture of the cytoplasmic membrane by eugenol may elucidate the translocation of macrocarpals into the cell, where they impede enzymatic activities and elevate ROS and intracellular DNA fragmentation (Tsukatani et al., 2020). Furthermore, eugenol showed a synergism effect with antimicrobial agents (fluconazole and azithromycin) against *C. albicans* and *S. mutans*. Eugenol can disrupt cell membrane integrity, facilitating the ingress of drugs into the microbial cell. This phenomenon facilitates the accessibility of the antimicrobial agents to the target site, enhancing their efficacy (Jafri et al., 2020). Therefore, the combined use of antimicrobial agents and eugenol may provide numerous benefits, such as increased efficacy, diminished medication dosage, and lowered toxicity, hence helping in the inhibition or eradication of biofilms and the reduction of antimicrobial resistance (Chouhan et al., 2017).

Nonetheless, light sensitivity and inadequate water solubility are significant drawbacks of eugenol, as they restrict its practical applications. *Sajomsang* et al., by incorporating eugenol into the β-cyclodextrin-grafted chitosan derivatives (QCD-g-CS) complex, developed a novel mucoadhesive drug carrier that could improve eugenol’s solubility, stability, and delivery properties while maintaining or enhancing its beneficial biological activities. The antibacterial efficacy of QCD-g-CS-eugenol against *Streptococcus oralis*, *C. albicans*, and *S. mutans* was assessed. Combining eugenol and QCD-g-CSs as an inclusion complex has a synergistic effect on antibacterial activity. The enhanced antibacterial efficacy of the synthesized drug carrier is ascribed to the augmented solubility of eugenol in the aqueous phase, facilitated by the presence of QCD-g-CS, thereby fostering improved interactions between eugenol and microorganisms (Sajomsang et al., 2012).

Finally, it is worth mentioning that studies showed the antibacterial effect of eugenol against *S. mutans* as a restorative material (Boeckh et al., 2002; Monajemzadeh et al., 2017; Makarla et al., 2023). Coronal leakage following root canal therapy is regarded as a significant cause of endodontic treatment failure due to contamination of the root canal system. Oral bacteria can infiltrate the root canal system during endodontic treatment without a coronal seal above the root filling. A base coronal to the root filling has been demonstrated to diminish microleakage and enhance the long-term prognosis of teeth treated with root canal therapy (Dragland et al., 2019). Nonetheless, microbial leakage has been demonstrated to occur even after installing temporary filling materials above the root filling (Balto, 2002). This issue indicates that the materials employed for a coronal seal must effectively occlude the opening of the root filling and exhibit antimicrobial characteristics. In this regard, a zinc oxide-eugenol (ZOE)-based restorative substance is among dentistry’s most frequently utilized temporary restorative materials. In endodontics, a ZOE restorative material serves as a base beneath the permanent restoration to inhibit bacterial ingress into the root canal between appointments and after the permanent restoration placement (Gimbel et al., 2002). The leaching of eugenol somewhat facilitates the antibacterial and bacteriostatic properties of ZOE-based materials (Dragland et al., 2019).

Collectively, eugenol, as a natural compound, exhibited good antibacterial and anti-biofilm activity against *S. mutans*, making it a suitable candidate for use in dentistry. Further research should also be considered to enhance its pharmacodynamic properties using novel methods, including nanoparticles and other drug delivery platforms.

## Farnesol

Farnesol, a sesquiterpene alcohol (3,7,11-trimethyl-2,6,10-dodecatrien-1-ol), is frequently present in certain essential oils, propolis, and citrus fruits and serves as a unique naturally occurring anticaries agent (Jeon et al., 2011). *Koo* et al. reported the MIC and MBC of this compound against *S. mutans* as 125 µM and 500 μM, respectively (Koo et al., 2002). A study’s results indicated that farnesol enhanced the initial rate of proton influx in *S. mutans* cells (Jeon et al., 2011). Protons from the external environment penetrate inward *via* the cell membrane following the acidification of the suspension but can subsequently be ejected by the membrane-associated F-ATPase enzyme (Bender et al., 1986). The proton-translocating F-ATPase safeguards *S. mutans* from ambient acid stress by maintaining pH homeostasis, which is essential for the optimal functioning of glycolysis in *S. mutans* (Sturr and Marquis, 1992). Consequently, the enhancement of proton permeability induced by farnesol is probably attributable to direct impairment of the membrane barrier function. The alteration of proton permeability in the *S. mutans* cell membrane caused by farnesol would impact the pH gradient (ΔpH) across the membrane, suppressing total intracellular metabolism, including acidogenesis (Jeon et al., 2011). Such effects may also induce energy deprivation in *S. mutans*, inhibiting intracellular polysaccharide (IPS) synthesis and accumulation (Jeon et al., 2009). It can be concluded that farnesol can influence the acidogenicity and aciduricity of *S. mutans* in both the planktonic phase and biofilm by enhancing the proton permeability of the membrane, possibly due to loss of the cell membrane’s functional integrity (Jeon et al., 2011).

Significant antibiofilm activity against *S. mutans* was shown by farnesol (Koo et al., 2002; Lobo et al., 2021). As previously noted, farnesol can disrupt membrane function, which reduces cell viability and could impact biofilm formation. A significant reduction in total biomass and the number of viable cells was observed in single and mixed species biofilms of *S. mutans* and *C. albicans* when exposed to farnesol (Fernandes et al., 2016). *De Melo* et al. reported the most significant reduction of *S. mutans* cells after 8 h of exposure to farnesol (de Melo et al., 2015). Notably, biofilms treated with farnesol exhibited a reduced quantity of viable cells and less dense structures. Moreover, the matrix composition study indicated a decrease in protein concentration following exposure to farnesol. These findings underscore the influence of farnesol on the diminution of biofilms, particularly its impact on the disintegration of the extracellular matrix. The extracellular matrix is significant as it constitutes a barrier to medication permeation (Fernandes et al., 2018).

Prior research has indicated that the reduction of long-chained cells constitutes the initial morphological alteration in the process of cell apoptosis, and this modification is also a mechanism of acid tolerance in *S. mutans* (Fozo and Quivey, 2004). To this end, the microscopic analysis revealed a reduced presence of long-chained cells in the farnesol-treated biofilms compared to the untreated control biofilms. The findings indicated that decreasing long-chained cells in the farnesol-treated biofilms may constitute a damaging mechanism of farnesol against *S. mutans* biofilm (Cao et al., 2017). Additionally, the transcription levels of *brpA*, *luxS*, *recA*, *ffh*, *smx*, and *nth* were decreased in the farnesol-treated biofilms (Cao et al., 2017). *Streptococcus mutans* employs a low-pH survival strategy by activating a DNA repair mechanism to safeguard or rectify DNA damage resulting from the detrimental effects of intracellular acidification. Certain proteins, including RecA, *N*th, Smx, and Ffh, participate in this process (Cao et al., 2017). Notably, RecA is implicated in stress tolerance and DNA repair. The *nth* gene encodes a putative EndoIII-related endonuclease implicated in DNA replication, recombination, and repair (Wen and Burne, 2004). A class II-like AP endonuclease that controls an organism’s exonucleolytic activity and acid-adaptive response is encoded by the *smx* gene (Faustoferri et al., 2005). The *ffh* gene encodes a homolog of the 54 kDa subunit of a signal recognition particle that contributes to acid stress tolerance (Gutierrez et al., 1999). Finally, the demineralization process induced by *S. mutans* biofilms diminishes enamel surface micro-hardness, resulting in caries lesions (Jabbarifar et al., 2011). The results indicated that farnesol therapy diminished the reduction of surface enamel micro-hardness caused by *S. mutans*, implying that farnesol may inhibit the development of caries associated with *S. mutans* and could serve as a possible agent against this bacterium (Cao et al., 2017). Therefore, farnesol can exert its anti-biofilm effect by disrupting stress and acid tolerance mechanisms, changing the expression of genes related to biofilm formation, and reducing the viable cells.

Farnesol has also demonstrated synergistic effects when combined with other anticaries agents (Koo et al., 2003; Chen et al., 2006; Lobo et al., 2022; Haj-Yahya et al., 2024). For example, *Rocha* et al. assessed the impact of farnesol, myricetin, and fluoride on dual-species biofilm, including *S. mutans* and *C. albicans*. The findings indicated a reduction in water-soluble EPS in the extracellular matrix when combination therapy was employed. Consequently, combination therapy adversely impacted biofilm growth, rendering biofilm potentially less hazardous. The reduction of EPS is crucial, as the capacity of bacteria to manufacture glucan may be more critical for virulence than their population size, given that a compromised extracellular matrix may fail to offer sufficient three-dimensional structure and stability for microorganisms within the biofilm (Rocha et al., 2018). Another study also investigated the effects of farnesol and apigenin in conjunction with fluoride on *S. mutans* biofilms. The biological effects of each drug were significantly amplified when combined with fluoride. Biofilms treated with farnesol and/or apigenin in conjunction with fluoride exhibited reduced biomass and diminished levels of iodophilic polysaccharides and insoluble glucans compared to those treated with the test agents individually. Findings from this research indicated that apigenin and farnesol may augment the cariostatic efficacy of fluoride (Koo et al., 2005).

In line with these results, farnesol, myricetin and fluoride combinations were examined against *S. mutans* biofilm (Falsetta et al., 2012). The mentioned combination therapy effectively diminished the formation of cariogenic biofilms *via* multiple potentially complementary and/or overlapping mechanisms that primarily focus on inhibiting *S. mutans* EPS-rich matrix production while undermining the organism’s overall fitness by suppressing stress defence and/or modifying bacterial membrane physiology. The mentioned combination therapy interfered with particular genes typically linked to EPS synthesis and/or stress resilience (e.g., *gtfB*, *sloA*, *sodA*, and *copY*). Notably, GtfB is essential for the formation and preservation of plaque biofilms. Superoxide dismutase (SodA) is a recognized virulence factor that diminishes superoxide and is crucial *in vivo* (Falsetta et al., 2012). SloA is a manganese/iron transport system component, whereas CopY is also anticipated to participate in copper transport (Mitrakul et al., 2004). The diminished expression of *sloA* and *copY* may further inhibit the transcription of the *gtf* genes, as copper and manganese function as effector molecules that regulate their expression (Chen et al., 2006; Arirachakaran et al., 2007). collectively, the mentioned combination therapies could successfully connect the existing chemical modalities (e.g., fluoride and chlorhexidine) employed to prevent or treat dental caries disease (Falsetta et al., 2012).

However, farnesol cannot attain optimal performance for biofilm therapy owing to its hydrophobic nature and inadequate biofilm retention unless utilized in conjunction with other methods (Rocha et al., 2018). Consequently, to attain optimal efficacy within the intricate biofilm community, drug delivery systems, particularly nanoparticle carriers, have garnered heightened interest in the treatment of oral biofilms in recent years (Horev et al., 2015; Mogen et al., 2015; Supuran, 2015; Sims et al., 2018; Barot et al., 2020; Roncari Rocha et al., 2022). A recently published study evaluated the anti-biofilm effectiveness of farnesol using drug delivery through polymeric nanoparticle carriers (NPCs) against cross-kingdom biofilms. The farnesol-encapsulated nanoparticles (NPC + Far) achieved a 2-log CFU/mL decrease of *S. mutans* and *C. albicans*. High-resolution confocal photos indicated a substantial reduction in EPS, smaller microcolonies of *S. mutans*, and the absence of hyphal forms of *C. albicans* following treatment with NPC + Far on human tooth enamel (HT) slabs, thereby modifying the biofilm’s three-dimensional structure (Ito et al., 2022). In another study, an NPC capable of co-loading farnesol in the hydrophobic core and myricetin inside the cationic corona was evaluated *in vitro*, employing both established and developing *S. mutans* biofilms. Co-loaded NPC treatments significantly diminished biofilm biomass and survival compared to single-drug controls in growing biofilms, indicating that dual-drug delivery demonstrates synergistic anti-biofilm properties. Co-loaded NPCs synergistically suppressed planktonic bacterial proliferation relative to controls and diminished *S. mutans* acidogenicity by lowering *atpD* expression (Sims et al., 2020). In another study, researchers developed a micellar drug delivery system capable of successfully adhering to dental surfaces. To attain tooth-binding capability, the terminal ends of biocompatible Pluronic copolymers were altered with a biomineral-binding group (i.e., alendronate). The micelles created with this polymer demonstrated the ability to rapidly (<1 min) adhere to hydroxyapatite (HA; a model tooth surface) and progressively release the encapsulated farnesol. *In vitro* biofilm inhibition investigations revealed that the synthesized micelles significantly inhibited *S. mutans* biofilm formation (Chen et al., 2009). Hence, drug delivery systems can be designed to enhance farnesol by prolonging the contact time with *S. mutans* to improve antibacterial effects. Moreover, these systems can enhance farnesol penetration into biofilms and increase access to bacteria embedded within the EPS matrix.

## Epigallocatechin-3-gallate

Epigallocatechin gallate (EGCG) is the predominant catechin in green tea, constituting about 50% of its overall composition. EGCG is recognized for possessing the most potent antibacterial properties among all catechins, according to the galloyl groups within its structure (Sasaki et al., 2004; Wang and Ho, 2009). Recently published studies reported that EGCG has an inhibitory effect against S. mutans (Bai et al., 2016; Hattarki et al., 2021; Garcia-Contreras et al., 2023; Higuchi et al., 2024). EGCG infiltrating lipid bilayers induces lateral membrane expansion, leading to membrane breakdown. Catechins can interact with dissolved oxygen to generate hydrogen peroxide and hydroxyl radicals, thereby causing intracellular lipid oxidation and damage to DNA and proteins (Wu and Brown, 2021). *Higuchi* et al. indicated that EGCG can suppress the metabolic activity of *S. mutans*. The authors hypothesized that the metabolic inhibition of this bacteria by EGCG is attributed to the binding of EGCG to the sugar uptake enzyme system, namely, the phosphoenolpyruvate phosphotransferase system (PEP-PTS), which leads to the inhibition of sugar absorption (Higuchi et al., 2024). In line with these findings, *Han* et al. also reported that EGCG can inhibit the growth and acid generation of *S. mutans* by suppressing the activity of the PEP-PTS (Han et al., 2021). Notably, the PEP-PTS is a cluster of enzymes responsible for translocating sugars into bacteria, comprising enzymes located on the cell membrane and within the cytoplasm. The results indicated that PEP-PTS activity was inhibited by EGCG, implying that EGCG obstructs glucose absorption in bacterial cells, hence diminishing bacterial metabolism and acidogenesis (Han et al., 2021). In a separate *in silico* molecular docking investigation, these authors indicated that catechins exert their effects through contact with the cell membrane-bound glucose transporter EIIC, a component of the PEP-PTS. In contrast to nongalloylated catechins, the galloyl structures of EGCG enabled strong binding to the functional region of the EIIC (Han et al., 2023). Furthermore, another research indicated that the inhibition of acid generation by EGCG is due to its suppressive effects on lactate dehydrogenase at both the transcriptional and enzymatic levels (Hirasawa et al., 2006; Xu et al., 2011).

In addition to the mentioned inhibitory mechanisms of EGCG against *S. mutans*, the results of another investigation indicated that EGCG impeded the adhesion of oral bacteria by diminishing their surface hydrophobicity; however, this effect was not observed on hydroxyapatite surfaces (Wang and Lam, 2020). Besides, *Cui* et al. demonstrated that EGCG caused aggregation in *S. mutans* (Cui et al., 2012). Therefore, EGCG can eliminate *S. mutans* by direct interaction with cellular components, and its inhibition of bacterial metabolism diminishes energy generation, possibly suppressing bacterial growth. Besides impeding metabolism and growth, EGCG obstructs bacterial adhesion and facilitates bacterial aggregation. Consequently, EGCG appears to be able to induce aggregation of salivary bacteria, facilitate their evacuation, and impede the formation of oral biofilm. Ultimately, EGCG can suppress the acid production of *S. mutans*, a main bacterial component in caries formation.

Previous studies also reported the inhibitory effects of EGCG against the *S. mutans* biofilm community (Liao et al., 2021; Taylor et al., 2021). To this end, a study indicated that EGCG markedly decreased the buildup of soluble and insoluble polysaccharides, leading to a biofilm characterized by irregularly distributed exopolysaccharide-microcolony complexes on enamel. Consequently, the authors suggested that EGCG diminished the pathogenicity of *S. mutans* matrix-rich biofilm by inhibiting the production of biofilm matrix constituents and modifying the structure, organization, and dispersion of the biofilm matrix (Aragão et al., 2024a). *Rayman* et al. also indicated that EGCG diminished biofilm thickness, lowered the viable bacterial count, augmented the number of deceased bacteria, and blocked EPS formation. EGCG markedly suppressed the expression of *gtfC*, *gtfB*, and *ftf* by 77%–90% relative to the control (Schneider-Rayman et al., 2021). The findings of other studies also showed that EGCG decreased levels of extracellular polysaccharides in *S. mutans* biofilms and reduced the expression of *gtf* genes (Xu et al., 2012; Wu et al., 2018).

EGCG appears to inhibit the biofilm formation of *S. mutans* through a sucrose-dependent anti-adhesion mechanism, as it has been demonstrated to obstruct *S. mutans* glucosyltransferases, which are critical enzymes for bacterial attachment, biofilm development, and pathogenicity in the presence of sucrose. In this context, *Islam* et al. indicated that EGCG interacted with the amino acids GLU 515 and TRP 517, binding to glucansucrase and inhibiting its enzymatic activity. Enzymatic suppression of glucansucrase reduced the biofilm-forming capability of *S. mutans* on tooth surfaces (Hairul Islam et al., 2020). Collectively, *S. mutans* produces extracellular adherent glucans from dietary sucrose through GTFs, hence facilitating the aggregation of oral bacteria on dental surfaces. The earliest phase of *S. mutans* biofilm, marked by sucrose-dependent bacterial adhesion to dental surfaces, constitutes a crucial preliminary step in the eventual development of the mature biofilm (Burne et al., 2009; Xu et al., 2011). To this end, EGCG has a good potential for inhibiting *S. mutans* biofilm because it inhibits initial attachment and the specific genes associated with bacterial biofilm formation.

Despite their advantages, catechins encounter significant obstacles in their development as medicinal agents, such as inadequate absorption, low bioavailability, and quick destruction. The advent of nanobiotechnology facilitates targeted and steady distribution, hence improving EGCG bioavailability and optimizing therapeutic efficacy. Recently published studies used different EGCG-based drug platforms, such as chitosan nanoparticles loaded with EGCG, mesoporous silica-based EGCG/nanohydroxyapatite delivery, developing silver nanoparticles (AgNPs) using EGCG, and EGCG containing glass ionomer cements (Hu J. et al., 2013; Yu et al., 2017; Yu et al., 2021; Aragão et al., 2024b). Noteworthy, using EGCG can enhance the inhibitory effect of different platforms against *S. mutans* and the biofilm community of this bacterium. The drug platform can also deliver EGCG over an extended period, ensuring prolonged stability to safeguard the underlying dentin from harmful conditions in oral settings. Consequently, applying EGCG-based pharmacological platforms indicates a feasible approach for prolonging the longevity of restorations and advancing dental material science.

## Thymol

*Thymus vulgaris* (thyme) has been utilized in traditional medicine to treat diverse diseases due to its extensive pharmacological qualities. The primary component of thyme essential oil is thymol, a phenolic monoterpene molecule (Priya et al., 2021). Thymol is a naturally occurring phenolic monoterpenoid compound that is utilized extensively in food and pharmaceutical preservative applications due to its beneficial antifungal and antibacterial qualities (Ferreira et al., 2018; Karimi et al., 2020; Priya et al., 2021). Recently published studies reported that thymol has an inhibitory effect against *S. mutans* (Botelho et al., 2007; Nunes et al., 2020; Park et al., 2023). For example, in one study, the MIC and MBC values of the phytochemical thymol against *S. mutans* were 312.5 μg/mL (Ferreira et al., 2018). In another investigation, thymol demonstrated bactericidal and bacteriostatic effects against the planktonic cells of *S. mutans*, with a MIC and MBC of 100 mg/mL and 400 mg/mL, respectively (Nunes et al., 2020). A recent study demonstrated that thymol at a 300 μg/mL dosage inhibited the growth of dual species of *S. mutans* and *C. albicans* (Priya et al., 2021). Cell membrane breakdown, intracellular substance leaking, and ensuing modifications in transmembrane potential are the foundations of the thymol mode of action. Additionally, this compound can enter cells and interact with intracellular locations, which is crucial to its antibacterial action (Vlachojannis et al., 2015). Furthermore, thymol can be incorporated into bacterial cell membranes, readily traverse lipid barriers, and compromise membrane integrity, thereby hindering cell proliferation and inducing cell death. Due to its lipophilic nature and affinity for bacterial cell membranes, thymol can inhibit bacterial activity (Yazdanian et al., 2022; Park et al., 2023). The genes of *S. mutans* associated with biofilm formation, competence, and glucan synthesis were downregulated by thymol (Priya et al., 2021). In line with these results, *Thymus* essential oil showed a notable inhibitory effect on the expression of the S. *mutans* virulence genes, including *brpA*, *vicR*, *gbpB*, *gtfB*, *gtfC*, *gtfD*, *relA*, and *spaP* (Table 2). Molecular docking between thymol and virulence proteins revealed that thymol demonstrated a significant binding affinity for the functional domains of virulence genes (Park et al., 2023).

**TABLE 2:** The molecular interactions of natural compounds with different pathogenic mechanisms of *Streptococcus mutans*.

Site of action	Compounds	Gene	Gene function
Environmental stress	Curcumin	*atpH*	It encodes subunit C of the F-ATPase, which is involved in the acid tolerance of *S. mutans*
		*spxA1*and *spxA2*	They regulate the transcription of nearly all principal activated oxidative stress response genes in *S. mutans*
	Cinnamaldehyde	*idh* and *recA*	DNA repair mechanisms
		*dnaK* and *atpD*	Regulate heat and acid-induced stress
		*nox1*	Oxidative stress tolerance
		*relA*	Synthesize (p)ppGpp
		*brpA*	Cell wall integrity
		*ciaH*	Stress tolerance
	Eugenol	*brpA* and *relA*	ME
	Thymol	*brpA* and *relA*	ME
	Carvacrol	*brpA* and *relA*	ME
	Farnesol	*brpA*	ME
		*recA*	Stress tolerance and DNA repair
		*nth*	DNA replication, recombination, and repair
		*smx*	Control of the organism’s exonucleolytic activity and the adaptive response to acid
		*ffh*	Acid stress tolerance
		*sodA*	Superoxide dismutase
Energy metabolism	Curcumin	*scrAB*	Internalization of sucrose and hydrolyze to fructose and glucose-6-phosphate
	Cinnamaldehyde	*pdhA, pdhB, pdhC,* and *pdhD*	They encodes subunits of the pyruvate dehydrogenase enzyme
		*atpD*	It encodes the β-subunit of the ATP synthase enzyme
		*eno, ldh,* and *pykf*	Glycolysis-related genes
	Farnesol	*atpD*	ME
Attachment and aggregation	Curcumin	*gtfB, gtfC,* and *gtfD*	Utilize sucrose and form glucan
		*gbpB*	It encodes glucan-binding proteins that bind to glucan
		*ftf* and *fruA*	Convert sucrose to the fructans
		*vicR*	A response regulator activates the *gtfB*, *gtfC*, and *ftf* genes
	Cinnamaldehyde	*gtfB, gtfC, gtfD, gbpB, vicR* and *ftf*	ME
		*spaP*	This gene mediates bacterial attachment to the salivary pellicle of the tooth
		*covR*	Regulate gtfB, gtfC, gtfD, and gbpC genes
	Eugenol	*gtfB, gtfC, gbpB, vicR, spaP* and *ftf*	ME
	Farnesol	*gtfB*	ME
		*sloA* and *copY*	They are involved in manganese/iron and copper transport, while these metals modulate the expression of *gtf* genes
	EGCG	*gtfB, gtfC,* and *ftf*	ME
	Thymol	*gtfB, gtfC, gtfD, vicR, gbpB,* and *spaP*	ME
	Carvacrol	*gtfB, gtfC, gtfD, vicR, gbpB,* and *spaP*	ME
Biofilm development	Curcumin	*rgpG*	Cell morphology and cell division
	Cinnamaldehyde	*atlA*	It encodes an autolysin enzyme that is essential for facilitating cell growth and division
		*ftsZ*	Cell division
		*gyrA*	DNA replication and maintenance of genomic stability
		*ciaH*	Sucrose-dependent biofilm formation
	Eugenol	*Smu630*	Biofilm formation hypothetical protein
Quorum sensing	Curcumin	*comCDE*	Intraspecies cell-cell communication
		*luxS*	Interspecies and intraspecies interaction
	Cinnamaldehyde	*comA, comE, comS, comR, comDE, comB, comEC* and *luxS*	ME
	Eugenol	*comDE*	ME
	Farnesol	*luxS*	ME

ME: mentioned earlier. EGCG: epigallocatechin gallate.

Scientists also considered thymol-based drug delivery platforms for improving thymol activity against *S. mutans* and dental caries. To this end, thymol-chitosan hydrogels demonstrated biocompatibility with [3T3] fibroblasts, exhibited antibacterial efficacy against *S. mutans* for 72 h, and showed antioxidant activity for 24 h. These are advantageous characteristics for a mucosal delivery method for an antimicrobial-antioxidant dual treatment targeting periodontal disease. Consequently, the antioxidant qualities of thymol may reduce periodontal inflammation; conversely, thymol could serve as an adjunct to mechanical plaque management due to its antibacterial efficacy. It should be noted that no adhesion or aggregation of *S. mutans* was detected in thymol-loaded chitosan hydrogels throughout a 24-h (Alvarez Echazú et al., 2017). Another study also nanoencapsulated the combination of clove oil and thymol (CLTY) utilizing chitosan and poly-γ-glutamic acid. Free CLTY demonstrated both additive and synergistic antibacterial effects against *S. mutans* and *Streptococcus sobrinus*, respectively. In contrast, in a time-kill kinetic experiment, CLTY nanoparticles (NPs) displayed synergistic action against both strains. CLTY NPs reduced the proliferation of salivary *S. mutans* during the evaluation, in contrast to free CLTY in the mouth rinse assay. The results demonstrated that nanoencapsulation can enhance the synergistic antibacterial efficacy of CLTY and prolong its antimicrobial activity in oral cavities (Lee et al., 2020). In the end, in another study, chitosan-grafted thymol (CST) coated on gold nanoparticles (AuNPs) was effectively employed to regulate cariogenic bacteria in the oral cavity. The incorporation of AuNPs with CST increased bactericidal efficacy against *S. mutans*. This CST coating on the AuNPs surface may represent a significant new tool in combating cariogenic bacterial infections (Chittratan et al., 2022). Collectively, the use of such compounds leads to the enhancement of thymols’ pharmacological activity and the expansion of its applications in medicine. Additionally, as mentioned, thymol demonstrated considerable inhibition of bacterial proliferation, acidogenesis, adhesion, and biofilm development of *S. mutans*. Therefore, because thymol is a natural anti-cariogenic agent with strong antibacterial effects against *S. mutans*, it can be used in oral and dental hygiene products and edible products such as chewing gum and candies.

## Carvacrol

Carvacrol, a phenolic monoterpenoid, is recognized as a principal component of the essential oils derived from several fragrant plants, including thyme (*Thymus vulgaris*), pepperwort (*Lepidium flavum*), and oregano (*Origanum vulgare*). This natural compound has been utilized as a food preservative, additive, flavouring, and scent in cosmetic items (Shariati et al., 2022). Carvacrol possesses multiple biological properties, such as antioxidant and antimicrobial activity. Due to its diverse qualities, including a free hydroxyl group, phenolic moiety, and hydrophobic characteristics, carvacrol demonstrated superior antibacterial efficacy compared to other volatile compounds. Several studies evaluated the antibacterial and antibiofilm effect of carvacrol, the results of which all confirm the antibacterial ability of this substance against *S. mutans* (Botelho et al., 2007; Ciandrini et al., 2014; Baygar et al., 2018; Pinna et al., 2019; Yazdanian et al., 2022). *Babiano* et al. assessed the antibacterial efficacy of carvacrol against pathogenic microorganisms responsible for oral infections, including *S. mutans* and *Streptococcus sanguinis*. The findings of their research indicated that sub-inhibitory concentration of carvacrol impeded bacterial growth. Furthermore, the death kinetics demonstrated a rapid and effective microbicide activity at doses that impact planktonic bacteria similarly to those shielded within their polymeric matrix. According to the results of morphological changes obtained from microscopic evaluation, the authors of this study explained the anti-biofilm effect of carvacrol as follows: the mechanism of action of carvacrol appears to be multifaceted, mostly contingent upon the molecular structural characteristics, which include a free hydroxyl group, a delocalized electron system, and hydrophobic properties. Carvacrol may target the cytoplasmic membrane, so affecting its development and functionality. Carvacrol’s hydrophobic characteristics enable it to interact with the lipid bilayer of the cytoplasmic membrane, positioning itself among the fatty acid chains. This interaction destabilizes the membrane structure, enhances fluidity, and increases permeability to potassium ions and protons, ultimately leading to cell death (Fernández-Babiano et al., 2022).

In line with this study, another experiment indicated that carvacrol showed significant bactericidal and antibiofilm properties against *S. mutans* and can serve as an eco-friendly alternative for managing dental caries. Anti-biofilm findings indicated that carvacrol diminished biofilm formation capability. The authors proposed that this compound contributes to the permeabilization and depolarization of the cytoplasmic membrane, reducing the pH gradient across the membrane. The reduction of the pH gradient disrupts the proton motive force, resulting in decreased intracellular ATP levels and, ultimately, cell death (Khan et al., 2017). In another study, thymus essential oils downregulated several genes encoding virulence factors associated with biofilm formation and maintenance, including *brpA*, *gbpB*, *gtfB*, *gtfC*, *gtfD*, *vicR*, *spaP*, and *relA* (Park et al., 2023). Collectively, results indicate that carvacrol exhibits antibacterial action against *S. mutans* and may be beneficial for preserving oral hygiene by inhibiting bacterial proliferation. Further investigations are recommended to enhance the understanding of this compound’s interaction with oral bacterial biofilms and its efficacy.

Finally, it is noteworthy that the interactions of other natural compounds with *S. mutans* are presented in Table 3.

**TABLE 3:** The inhibitory effect of natural compounds against *Streptococcus mutans* and the biofilm community of this bacterium.

Natural compounds	Source	Antibacterial concentration	Outcomes	References
1,8-Cineole	Eucalyptus globulus	1.9168 mg/mL	The EOs showed antimicrobial activity against the *S. mutans* planktonic and biofilm cultures	Landeo-Villanueva et al. (2023)
1,8-Cineole	Vitex agnus-castus leaves (VAC-EO)	15.6 µg/mL	VAC-EO indicated promising activity against *S. mutans*	Gonçalves et al. (2017)
1,8-Cineole (Eucalyptol)	Eucalyptus globulus	1.9168 mg/mL	Antibacterial and antibiofilm effect	Landeo-Villanueva et al. (2023)
Apigenin	Extrasynthese Co. (Genay-Sedex, France)	0.1 mM	This compound modulated the genetic expression of virulence factors in *S. mutans*	Koo et al. (2006)
Apigenin	Extrasynthese Co. (Genay-Sedex, France)	500 μM	Apigenin is a novel and potent inhibitor of GTF activity	Koo et al. (2002)
Apigenin and tt-Farnesol	Brazilian propolis	NR	Incorporating chemicals into resin-based composites and cement materials can markedly reduce the biomass and polysaccharide content of an *S. mutans* biofilm	André et al. (2021)
Apigenin and tt-Farnesol	Sigma–Aldrich	Apigenin (1 mM) and tt-Farnesol (5 mM)	Addition of the compounds to the self-etch adhesive) and to the each-and-rinse adhesive reduced the dry-weight of *S. mutans* biofilm.	André et al. (2017)
Carvone	Mentha spicata	1.8484 mg/mL	Antibacterial and antibiofilm effect	Landeo-Villanueva et al. (2023)
Coumaric acid	Sigma Aldrich	NR	Antibacterial and antibiofilm effect	Ahmad et al. (2024)
Ellagic acid	Rubi Fructus extract	<1 mg/mL	This compound inhibited glucosyltransferase activity of *S. mutans*	Ham and Kim (2023)
Ellagic acid	Sigma–Aldrich	500 μg/mL	The enzymatic activity of the glucosyltransferases of *S. mutans* was shown to be abrogated by ellagic acid and its derivatives	Sawamura et al. (1992)
Hinokitiol, Carvacrol, Thymol, Menthol	Sigma-Aldrich	40, 400, 200, 1,000 μg/mL, respectively	Antibacterial effect against *S. mutans*	Wang et al. (2016)
Kaempferol	*Phytolacca americana*	8 μg/mL	Kaempferol exerted antibacterial activity against *Porphyromonas gingivalis and S. mutans*	Patra et al. (2014)
Kaempferol	*Nidus Vespae* chloroform/methanol extraction	1 mg/mL	This compound inhibited the growth, acidogenicity and acidurity of *S. mutans*	Guan et al. (2012)
Kaempferol	*Nidus Vespae*	8 mg/mL	This compound showed Aantibiofilm activity	Zeng et al. (2019)
Limonene and β-caryophyllene	*Psidium guajava*	0.05–0.1%	Antimicrobial activity against *C. albicans* and *S. mutans*	Alam et al. (2023)
Linalool	Quinari	1,250 μg/mL	Antibacterial effect against *S. mutans*	Ferreira et al. (2018)
Linalool	Achillea ligustica	310 µg/mL	Antibacterial effect against *S. mutans*	Maggi et al. (2009)
Linoleic acid	Dryopteris crassirhizoma	12.5 µg/mL	Reduced viability in a dose-dependent manner and reduced biofilm accumulation during initial and mature biofilm formation	Jung et al. (2014)
Myrcene	Cymbopogon citratus	No effect	Myrcene did not show bacteriostatic activity at tested concentrations	Chaves-Quirós et al. (2020)
Myricetin	MolPort ordering service	250 μg/mL	The compound decreased the counts of *S. mutans* viable population by > 4 logs and biomass by >99%	Castillo Pedraza et al. (2020)
Myricetin	Sigma–Aldrich	512 μg/mL	Myricetin is a promising candidate for controlling dental caries and reducing *S. mutans* biofilm	Hu et al. (2021)
Myricetin	AK Scientific, Inc.	NR	The compounds resulted in reduced amounts of insoluble dry weight and insoluble exopolysaccharides	Lopes et al. (2022)
Myricetin and tt-farnesol	Genay-Sedex, France and Sigma-Aldrich	NR	The combination of compounds with fluoride significantly impeded the expression of particular virulence genes, structural organization and accumulation of *S. mutans* biofilm	Jeon et al. (2009)
Quercetin	*Nidus Vespae*	16 mg/mL	This compound showed antibiofilm activity	Zeng et al. (2019)
Quercetin	*Nidus Vespae*	2 mg/mL	This compound inhibited the growth of different bacteria, such as *S. mutans*	Guan et al. (2012)
Quercetin	*Cucumis sativus* peels	NR	The compound inhibited the growth of different bacteria, such as *S. mutan*s	Anjani et al. (2023)
Quercetin-doped adhesive	Sigma–Aldrich	500 μg/mL	Inhibitory effect against *S. mutans* biofilm	Yang et al. (2017)
Resveratrol	Bulksupplements, United States	250 μg/mL	This compound showed Aa dose-dependent antibacterial activity against *S. mutans*	Pournasir et al. (2024)
Resveratrol	Sigma-Aldrich	800 μg/mL	This compound showed has an inhibitory effect against *S. mutans* acid production, virulence factors and biofilm formation	Li et al. (2020b)
Resveratrol-doped adhesive	Sigma-Aldrich	1 mg/mL	Inhibitory effect on endogenous protease activity and biofilm formation of *S. mutans*	Guo et al. (2021)
Sanguinarine, eucalyptol, menthol, methyl salicylate	NR	15.6, 250, 500, and 1,000 μg/mL, respectively	Antibacterial effect against *S. mutans*	Chung et al. (2006)
Saponin	Madhuca longifolia and Bauhinia purpurea	18.3 and 890 µg/mL	Antibacterial effect against *S. mutans*	Jyothi and Seshagiri (2012)
Terpenoid	Myrmecodia pendans	40 µg/mL	Antibacterial and antibiofilm effect	Gartika et al. (2018)
Terpinen-4-ol	Sigma-Aldrich	0.24% µg/mL	Antibacterial and antibiofilm effect and modulated gene expression	Bordini et al. (2018)
Terpinen-4-ol	Sigma-Aldrich	44,000 μg/mL	Antibacterial effect against *S. mutans*	Bucci et al. (2018)
Ursolic acid and Oleanolic acid	Sigma-Aldrich	256 and 1,024 μg/mL, respectively	The compounds suppressed the growth of *S. mutans* and the biofilm community of this bacterium	Zhou et al. (2013)
α- phellandrene	Most ingredients of hydroxylated sesquiterpenes in the Piper barbatum kunth leaves EO	132 μg/mL	The compound showed an antibacterial effect against different microorganisms such as *S. mutans*	Noriega et al. (2020)
Terpinen-4-ol (the major component, 26.3%)	Lindera caesia	250 µg/mL	Potential in dental application for caries prevention	Zaini et al. (2023)
Camphor (the major component, 18.9%)	Rosmarinus officinalis	1,500 µg/mL	The EOs displayed low activity against the selected microorganisms. The pure major compounds were more active than the EO.	Bernardes et al. (2010)
Myrcene (the major component, 59%)	Protium heptaphyllum	50 µg/mL	The leaf EOs displayed very promising activity against *S*. *mutans* and *Streptococcus mitis*	Cabral et al. (2020)

## Conclusion

Recently published studies have focused on the role of microorganisms in the pathogenesis of caries, but oral bacterial resistance to newly developed antibacterial drugs is a newer concern. Hence, it is urgent to develop new drugs that inhibit bacterial QS and biofilm formation for antibacterial to prevent dental caries. Due to the increasing microbial resistance to existing antibiotics and the decline in the development of new drugs, global interest in natural antiseptic products derived from medicinal plants has grown. Previous studies have shown that natural products are promising for developing new anti-caries materials. In this review, natural compounds showed inhibitory effects on *S. mutans*, one of the primary causative agents of dental caries. These compounds reduced the planktonic population of this bacterium and had a remarkable effect on its biofilm population. However, these compounds have poor pharmacodynamic properties. Hence, novel drug delivery platforms can enhance their efficacy and productivity. In addition, since *S. mutans* alone does not cause caries and it develops caries with the help of other microorganisms, future studies should be more focused on investigating multi-species biofilms and optimizing the delivery methodologies of these compounds. Given the promising effects of these compounds, they could be used as a supplement alongside conventional anti-caries drugs such as fluoride or chlorhexidine. Combining these compounds with other antimicrobial agents may lead to synergistic effects and, in addition to enhancing overall efficacy, could reduce the required dosage of conventional drugs and possible side effects. Finally, although laboratory studies are valuable, their results must be validated *in vivo* and clinical settings to ensure their practical application in prevention and treatment.
