# Supplementary material for: Natural compounds: new therapeutic approach for inhibition of Streptococcus mutans and dental caries
Source: Front Pharmacol. 2025 Apr 1;16:1548117. doi: 10.3389/fphar.2025.1548117 (PMC11996897; doi:10.3389/fphar.2025.1548117)
Supplement: Supplementary file 1 [file Table1.docx]

**Table S1.** The source and chemical structure of natural compounds.

| **Natural compound** | **Source** | **Chemical Structure** **Depiction** | **Reference** |
| --- | --- | --- | --- |
| **Curcumin** | *Turmeric (Curcuma longa)* | 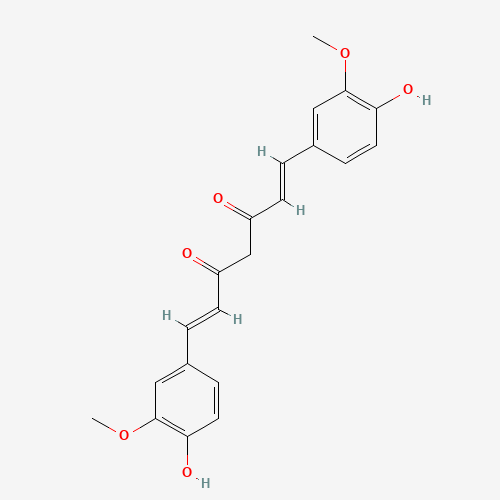 | (National Center for Biotechnology Information (2025). PubChem Compound Summary for CID 969516) |
| **Cinnamaldehyde** | Cinnamon bark | 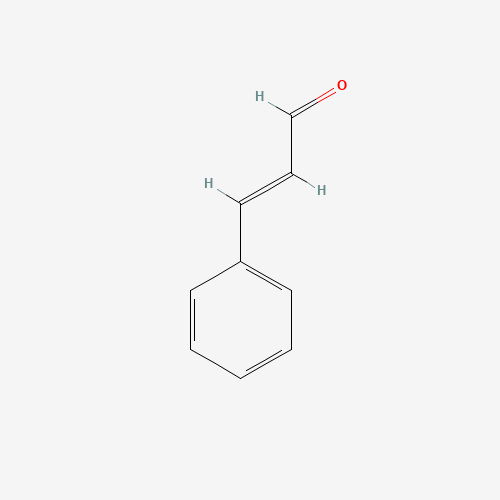 | (National Center for Biotechnology Information (2025). PubChem Compound Summary for CID 637511) |
| **Eugenol** | Clove buds,  Cinnamon bark and leaves,  Tulsi leaves,  Turmeric,  Pepper,  Ginger,  Oregano, and thyme | 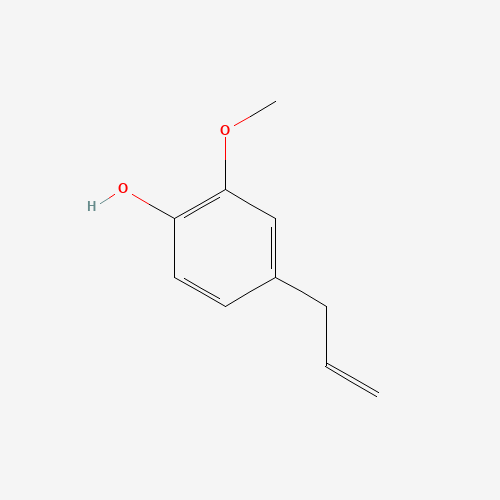 | (National Center for Biotechnology Information (2025). PubChem Compound Summary for CID 3314;Khalil et al., 2017) |
| **Farnesol** | Propolis,  Citrus fruits,  Citronella (*Cymbopogon nardus*),  Lemon grass (*Cymbopogon citratus*),  Tuberose (*Polianthes tuberosa L*),  Cyclamen (*Cyclamen persicum*),  Rose (*Rosa hybrida*),  Balsam neroli (*Citrus* *aurantium*), and Musk (*Abelmoschus moschatus*) | 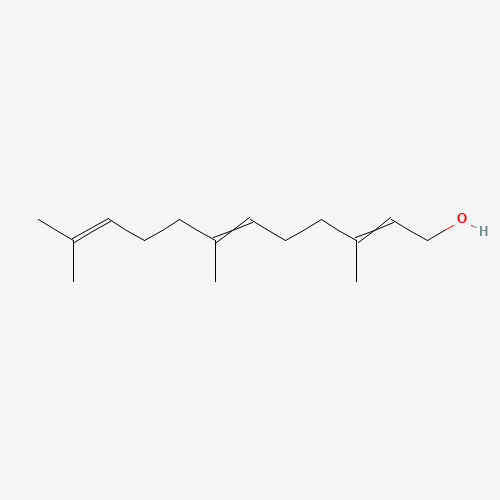 | (National Center for Biotechnology Information (2025). PubChem Compound Summary for CID 3327;Jung et al., 2018) |
| **Epigallocatechin gallate** | Green tea,  White tea, and  Black tea | 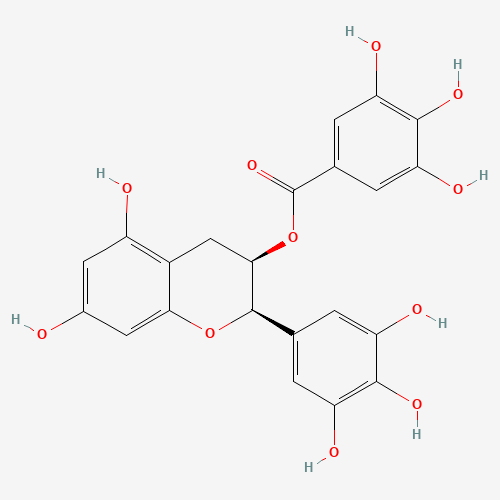 | (National Center for Biotechnology Information (2025). PubChem Compound Summary for CID 65064) |
| **Thymol** | *Thymus vulgaris* | 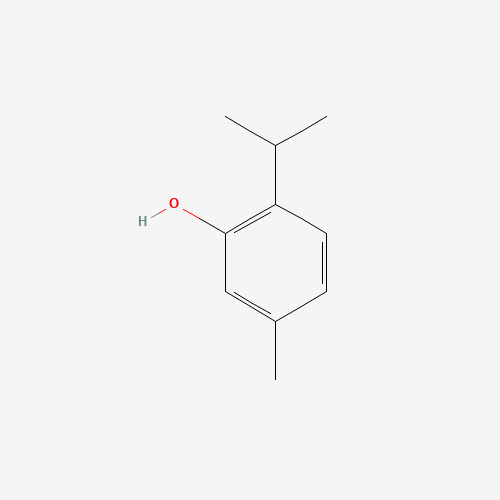 | (National Center for Biotechnology Information (2025). PubChem Compound Summary for CID 6989) |
| **Carvacrol** | Thyme (*Thymus vulgaris*),  Pepperwort (*Lepidium flavum*),  Oregano (*Origanum vulgare*),and  Wild bergamot (*Citrus aurantium bergamia*) | 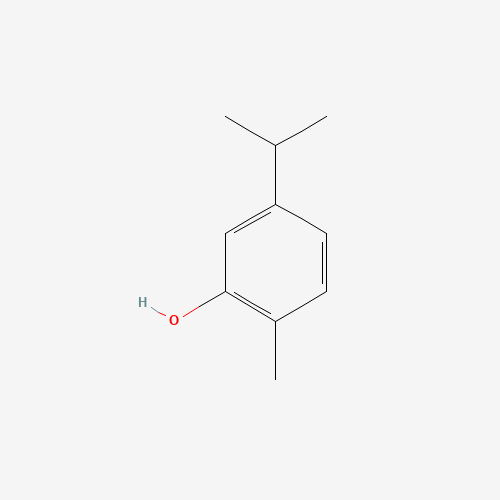 | (National Center for Biotechnology Information (2025). PubChem Compound Summary for CID 10364;Sharifi-Rad et al., 2018) |

**References**

Jung, Y.Y., Hwang, S.T., Sethi, G., Fan, L., Arfuso, F., and Ahn, K.S. (2018). Potential Anti-Inflammatory and Anti-Cancer Properties of Farnesol. *Molecules* 23(11). doi: 10.3390/molecules23112827.

Khalil, A., Rahman, U., Khan, M., Sahar, A., Mehmood, T., and Khan, M. (2017). "Essential oil eugenol: sources, extraction techniques and nutraceutical perspectives. RSC Adv 7: 32669–32681".).

National Center for Biotechnology Information (2025). Pubchem Compound Summary for Cid 3314, E.R.J., 2025 from <Https://Pubchem.Ncbi.Nlm.Nih.Gov/Compound/Eugenol>. doi:

National Center for Biotechnology Information (2025). Pubchem Compound Summary for Cid 3327, F.R.J., 2025 from <Https://Pubchem.Ncbi.Nlm.Nih.Gov/Compound/Farnesol>. doi:

National Center for Biotechnology Information (2025). Pubchem Compound Summary for Cid 6989, T.R.J., 2025 from <Https://Pubchem.Ncbi.Nlm.Nih.Gov/Compound/Thymol>. doi:

National Center for Biotechnology Information (2025). Pubchem Compound Summary for Cid 10364, C.R.J., 2025 from <Https://Pubchem.Ncbi.Nlm.Nih.Gov/Compound/Carvacrol>. doi:

National Center for Biotechnology Information (2025). Pubchem Compound Summary for Cid 65064, E.G.R.J., 2025 from <Https://Pubchem.Ncbi.Nlm.Nih.Gov/Compound/Epigallocatechin-Gallate>. doi:

National Center for Biotechnology Information (2025). Pubchem Compound Summary for Cid 637511, C.R.J., 2025 from <Https://Pubchem.Ncbi.Nlm.Nih.Gov/Compound/Cinnamaldehyde>. doi:

National Center for Biotechnology Information (2025). Pubchem Compound Summary for Cid 969516, C.R.J., 2025 from <Https://Pubchem.Ncbi.Nlm.Nih.Gov/Compound/Curcumin>. doi:

Sharifi-Rad, M., Varoni, E.M., Iriti, M., Martorell, M., Setzer, W.N., Del Mar Contreras, M., Salehi, B., Soltani-Nejad, A., Rajabi, S., Tajbakhsh, M., and Sharifi-Rad, J. (2018). Carvacrol and human health: A comprehensive review. *Phytother Res* 32(9)**,** 1675-1687. doi: 10.1002/ptr.6103.
